# Assessment of Yellow Fever Epidemic Risk: An Original Multi-criteria Modeling Approach

**DOI:** 10.1371/journal.pntd.0000483

**Published:** 2009-07-14

**Authors:** Sylvie Briand, Ariel Beresniak, Tim Nguyen, Tajoua Yonli, Gerard Duru, Chantal Kambire, William Perea, The Yellow Fever Risk Assessment Group (YF-RAG)

**Affiliations:** 1 Epidemic and Pandemic Alert and Response, World Health Organization, Geneva, Switzerland; 2 Liraes, Paris-Descartes University, Paris, France; 3 Institut Bioforce Développement Afrique, Bobo-Dioulasso, Burkina Faso; 4 Claude-Bernard University, Villeurbanne, France; 5 World Health Organization Regional Office for Africa, Country Office, Ouagadougou, Burkina Faso; Universidade de São Paulo, Brazil

## Abstract

**Background:**

Yellow fever (YF) virtually disappeared in francophone West African countries as a result of YF mass vaccination campaigns carried out between 1940 and 1953. However, because of the failure to continue mass vaccination campaigns, a resurgence of the deadly disease in many African countries began in the early 1980s. We developed an original modeling approach to assess YF epidemic risk (vulnerability) and to prioritize the populations to be vaccinated.

**Methods and Findings:**

We chose a two-step assessment of vulnerability at district level consisting of a quantitative and qualitative assessment per country. Quantitative assessment starts with data collection on six risk factors: five risk factors associated with “exposure” to virus/vector and one with “susceptibility” of a district to YF epidemics. The multiple correspondence analysis (MCA) modeling method was specifically adapted to reduce the five exposure variables to one aggregated exposure indicator. Health districts were then projected onto a two-dimensional graph to define different levels of vulnerability. Districts are presented on risk maps for qualitative analysis in consensus groups, allowing the addition of factors, such as population migrations or vector density, that could not be included in MCA. The example of rural districts in Burkina Faso show five distinct clusters of risk profiles. Based on this assessment, 32 of 55 districts comprising over 7 million people were prioritized for preventive vaccination campaigns.

**Conclusion:**

This assessment of yellow fever epidemic risk at the district level includes MCA modeling and consensus group modification. MCA provides a standardized way to reduce complexity. It supports an informed public health decision-making process that empowers local stakeholders through the consensus group. This original approach can be applied to any disease with documented risk factors.

## Author Summary

This article describes the use of an original modeling approach to assess the risk of yellow fever (YF) epidemics. YF is a viral hemorrhagic fever responsible in past centuries for devastating outbreaks. Since the 1930s, a vaccine has been available that protects the individual for at least 10 years, if not for life. However, immunization of populations in African countries was gradually discontinued after the 1960s. With the decrease in immunity against YF in African populations the disease reemerged in the 1980s. In 2005, WHO, UNICEF, and the GAVI Alliance decided to support preventive vaccination of at-risk populations in West African endemic countries in order to tackle the reemergence of YF and reduce the risk of urban YF outbreaks. Financial resources were made available to scale up a global YF vaccine stockpile and to support countries with limited resources in the management of preventive vaccination campaigns. This article describes the process we used to determine the most at-risk populations using a mathematical model to prioritize targeted immunization campaigns. We believe that this approach could be useful for other diseases for which decision making process is difficult because of limited data availability, complex risk variables, and a need for rapid decisions and implementation.

## Introduction

After several decades of relative calm, yellow fever (YF) outbreaks have had a resurgence in Africa, posing an immediate risk to the affected populations across the continent. Increasing migration, accelerating urbanization, and improved travel infrastructure are global trends that increase the risk of YF spreading to parts of the world where the disease has disappeared, such as Europe or North America, or never seen before, such as Asia [1]–[14]. Because of the risk of international spread, YF is one of the diseases officially reported under the International Health Regulations. The continued use of the YF vaccination certificate is a tangible sign of the constant threat posed by the disease at a global level [15]–[20].

The most effective measure for preventing and controlling YF outbreaks is vaccination. The development of a YF vaccine in the 1930s was a turning point in the history of the disease, because a single dose of the vaccine that is considered safe and effective is sufficient to protect an individual for at least 10 years and probably up to 35 years [21]–[24]. Between 1933 and 1961, mass vaccination campaigns were carried out in several francophone West African countries, resulting in the rapid disappearance of the disease over the subsequent 40 years [25]–[27]. The mass vaccination campaigns stopped in the 1960s when the French neurotropic vaccine (FNV) stopped being recommended for children under 10 years old because of the noted association with a high incidence of encephalitis reaction in this age group [28],[29]. The production of FNV stopped in 1980. Today the 17D vaccine is the only type of YF vaccine produced and used for vaccination.

While anglophone countries such as Nigeria experienced devastating YF outbreaks in the 1980s, francophone countries reported limited YF outbreaks. These outbreaks mainly appeared in nomadic communities or among seasonal workers (e.g., in Senegal 1965 and Burkina Faso 1983) who did not benefit from previous mass vaccination campaigns [30]–[34].

Since 2000, a number of YF outbreaks have been reported in West African countries, especially in capitals and large cities [35]–[40] such as Abidjan, Ivory Coast (2001); Dakar, Senegal (2002); Touba, Senegal (2002); Conakry, Guinea (2002); and Bobo Dioulasso, Burkina Faso (2004). The outbreaks were rapidly controlled by emergency reactive vaccination campaigns, and the number of YF cases has remained low.

The resurgence of this disease is related to a high proportion of non-protected individuals in exposed communities. The YF vaccine has been introduced into routine infant immunization programs in 19 of the 23 (83%) high-risk African countries endemic for YF [41]. However, with routine immunization of children alone, it takes several decades to reduce significantly the proportion of non-immune people in the population and thus the risk of outbreaks. Four strategies proposed by WHO–UNICEF have the potential to bring YF under control in Africa: (i) Rapid response to outbreaks, (ii) routine childhood immunization, (iii) mass preventive campaigns, and (iv) improved surveillance. Unfortunately, in most of the YF endemic countries, coverage for routine YF immunization is low (below 60%) and preventive campaigns have not been carried out programmatically. The limited implementation of recommended control strategies is due to many factors, including competing public health priorities such as meningitis or cholera outbreaks, the cost of the vaccination campaigns, and limited availability of affordable YF vaccine on the global market. The resurgence of YF is also linked to the interaction of various environmental, economic, social, and political factors. All these factors and their interactions make YF epidemic risk analysis a complex and difficult process: it requires an assessment of multiple criteria [42].

In the face of the resurgence of YF, the Global Alliance for Vaccine and Immunization (GAVI) has funded a joint WHO–UNICEF proposal in December 2005 to reduce YF epidemic risk in the following 12 high-risk countries in Africa: Benin, Burkina Faso, Cameroon, Côte d'Ivoire, Ghana, Guinea, Liberia, Mali, Nigeria, Senegal, Sierra Leone, and Togo. This initiative of US$62 million is aimed at providing sufficient funds by 2010 for the immunization of 48 million people, which represents approximately 17% of the population of the 12 targeted countries. In order to best use this limited international funding it is important to define levels of risk for YF outbreaks and to target high-risk communities for priority vaccination.

This article describes the process, methodology, and tools used to identify high-risk populations for priority vaccination, and the multiple correspondence analysis (MCA) and consensus assessment at the country level used as decision-making tools.

## Methods

### Overall risk assessment structure

The frame of reference chosen to identify communities at highest risk is derived from the model of Sutherts to assess the vulnerability of the population to vector-borne diseases. The vulnerability may be defined as the economic, social, or political predisposition of a community to destabilization by an external, natural, or man-made phenomenon [43]. YF vulnerability depends on three parameters: susceptibility of the community to infection, exposure to the YF virus, and resilience of the population at risk [26],[44].
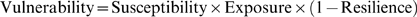



The susceptibility to or likelihood of a community being affected by a YF outbreak depends on population immunity, which is mainly related to the proportion of vaccinated people in a community. Susceptibility usually varies among countries and districts. Since the reemergence of YF in Africa, affected countries have embarked on preventive immunization campaigns or epidemic response campaigns, and have incorporated YF vaccine into the routine infant immunization schedule. Evidence suggests that epidemic risk in a community diminishes considerably once 60%–80% of the population in that community has been immunized [45]–[47]. Since mass vaccination campaigns may not reach 100% of the population, sporadic cases in a vaccinated population can still occur, but transmissions rate will remain low and will not amplify into epidemic transmission. Sporadic cases are also seen in non-immunized migrants settling in areas with infected mosquitoes.

The exposure is defined by the likelihood for a community to be in contact with the YF virus through infected *Aedes* mosquitoes. Resilience is the ability to control and recover quickly from an outbreak, and it depends on the capacity to quickly detect outbreaks and rapidly launch mass vaccination campaigns.

### Development of the risk assessment tool

The final product was a district-level assessment of vulnerability within countries. The assessment was derived from two processes, one quantitative and one qualitative. The quantitative assessment was based on the selection of key variables followed by a formal MCA [48]–[54]. This process led to a graphic representation of districts' vulnerability profile, allowing the definition of vaccination priorities according to the profile of vulnerability. The qualitative process consisted of consensus expert groups meeting at the country level that reviewed the results from the quantitative assessment and adjusted the districts' classifications based on additional information available at the country level. These groups consisted of various experts in the fields of epidemiology, virology, entomology, and public health from Ministries of Health, WHO, UNICEF, Institut Pasteur, and international nongovernmental organizations (NGOs).

### Quantitative assessment

#### Selection of variables

YF risk factors fall into three main categories: human, mosquito, and animal host, additionally influenced by the climate and ecological environment. The selection of relevant factors and related variables was done by an international expert panel. It was based on two sets of criteria (i) the relevance to the objective of the assessment, which is to identify populations to be vaccinated in priority; and (ii) availability and quality of data in all counties included in the initiative. We sought to identify factors based on data availability—which varies considerably across the 12 identified African countries—to facilitate the comparability of data between populations. Given the urgency of providing information to guide vaccination campaigns, we considered only currently existing data or data that could be easily collected without carrying out additional surveys. This approach was pilot tested in Burkina Faso to determine the feasibility and accuracy of the risk assessment tool before it was used in the remaining 11 countries targeted by the YF initiative. All data were gathered at the health district level in collaboration with the Ministries of Health and the WHO country office.

**Figure 1:**
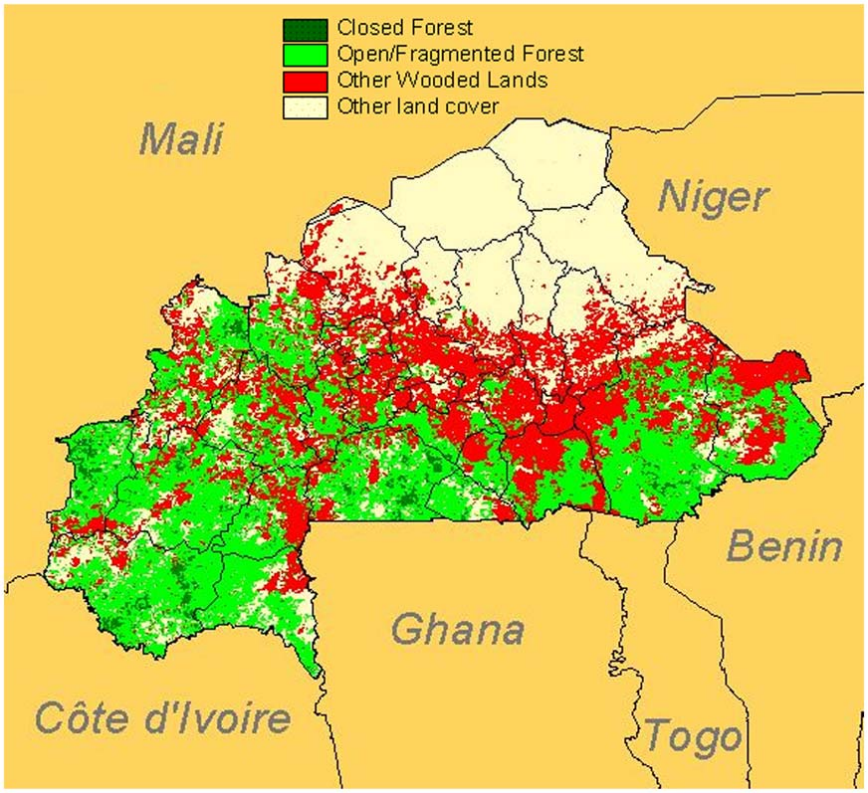
District situated in the ecological risk zone 15°N–10°S, wet savannah or dry forest (Source: FAO).

**Figure 2:**
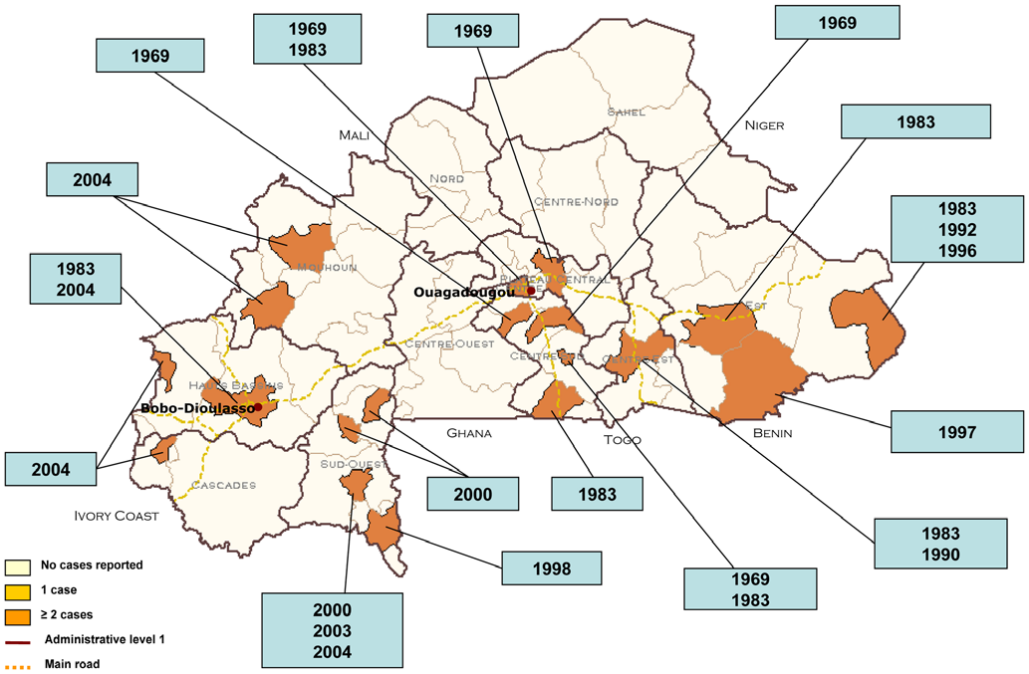
Yellow fever cases reported in Burkina Faso from 1950 to 2004.

The expert panel selected six indicators that were collected in the pilot country. Five “exposure-related” indicators were identified:

1. District situated in the ecological risk zone 15°N–10°S, wet savannah or dry forest [22]: Yes/No response (see Figure 1)
2. District reporting confirmed cases since 1960: Yes/No response (see Figure 2)
3. District reporting suspected cases between 1960 and the establishment of surveillance based on laboratory-confirmed cases: Yes/No response
4. Number of years in which any YF cases were reported since 1960 in this district
5. District close to another district that had any reported cases since 1960: Yes/No response

Modeling with multiple correspondence analysis (described in more detail below) was used to reduce the five exposure variables into a single aggregated exposure indicator.

The second indicator was a “community susceptibility” indicator, which represents the proportion of non-immunized persons in the district. This information was obtained by subtracting the number of persons already immunized from the total health district population. The number of vaccinated persons consisted of all vaccinated children and adults, vaccinated through either preventive or epidemic response campaigns, plus the number of infants immunized under the routine immunization program for the last 10 years. These numbers were obtained through Ministries of Health administrative vaccination coverage data.

Indicators related to resilience were too numerous and various in countries for inclusion in the quantitative process. This important part of the analysis was therefore kept for the qualitative assessment. The quantitative assessment of vulnerability thus was defined by the aggregated exposure indicator and the susceptibility indicator.

#### Multiple correspondence analysis

MCA is a descriptive technique developed in the 1970s by Jean-Paul Benzécri [48]. Although mostly used in socio-economic research it allows the analysis of any kind of complex matrix [51],[54]. MCA belongs to a group of multiple-criteria analysis techniques including a range of similar modeling techniques, such as factor analysis, cluster analysis, segmentation analysis, and neuronal network modeling [49], [55]–[57]. MCA was selected for the YF risk assessment for the following reasons: it is reproducible, results are understandable by national health authorities, it can address quantitative and qualitative variables, and it has the potential to generate a “quality indicator” to assess the robustness of the outcome.

For the current risk assessment, the five exposure variables were placed in columns and health districts in rows. MCA was used to “aggregate” the various exposure variables into a single aggregated exposure indicator. Usually MCA is used to project initial variables in a “factorial graph” composed on two axes. In this risk assessment, we have programmed a specific MCA, which allowed the projection of the five exposure variables into one single axis (exposure axis).

The interpretation of the factorial graph is much easier than the interpretation of the matrix, namely: the closer the districts lie together in the factorial graph, the more similar they are in terms of level of exposure.

Many professional statistical software tools include standard MCAs. However, no marketed package allows selection of the axis that would best synthesize risk factors and projection of multi-dimensional data on this single axis. This is the reason we have specifically programmed for this project a MCA tool that can manage large matrix calculations and allows the construction and the interpretation of one single-dimension factorial graph.

**Figure 3:**
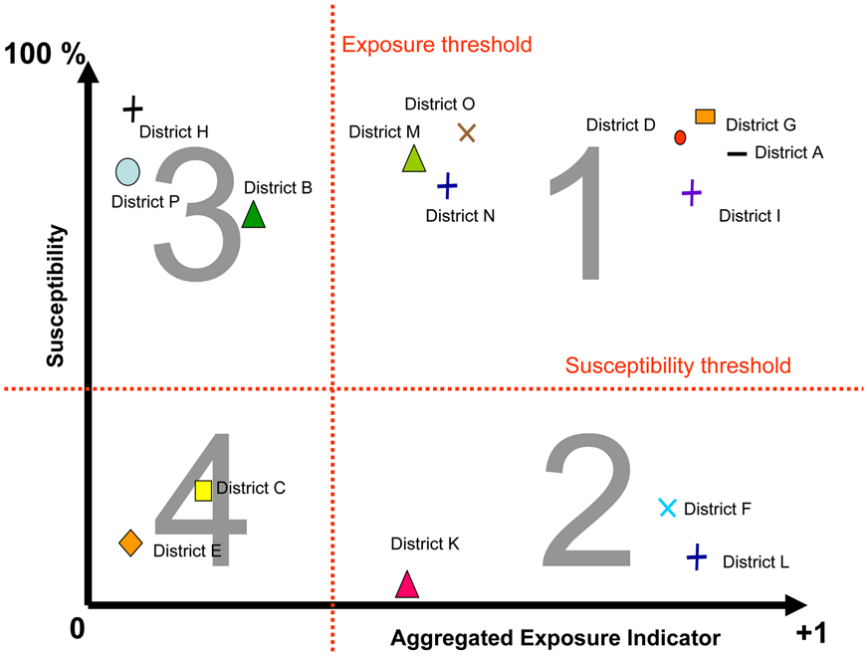
Yellow fever vulnerability model for decision making.

A vulnerability graph was built assigning the susceptibility variable to the *y*-axis and the aggregated exposure indictor to the *x*-axis (see Figure 3). The vulnerability profile of each district depends on its position on the graph, defined by the susceptibility value and the exposure value

Four profiles were defined on this graph based on the susceptibility and exposure threshold. The susceptibility threshold was set at 40%, indicating that >60% of the population has been immunized and that the risk of an epidemic is lower [46],[58]. The exposure threshold was discussed during the consensus meeting and is specific to each country. There is no absolute threshold value for exposure, because the value of the aggregated exposure indicator was relative to the group of districts considered in the analysis.

**Figure 4:**
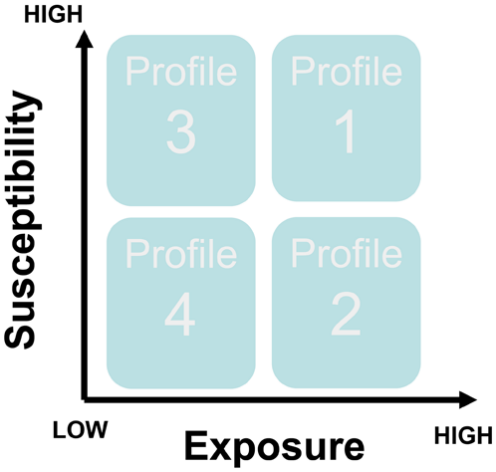
Yellow fever vulnerability profiles of districts identified with the quantitative assessment.

As noted above, the quantitative assessment profiles four clusters of districts characterized as follows (see Figure 4):

Profile 1: High exposure+high susceptibility: Very vulnerable, high priority for vaccination.
Profile 2: High exposure+low susceptibility: Vulnerable but no preventive vaccination in coming years because the population has already been immunized in the past 5 years.
Profile 3: Low exposure+high susceptibility: Vulnerable but lower risk than districts in profile 1.
Profile 4: Low exposure+low susceptibility: Low vulnerability and lower priority than districts in profile 2.

The analysis was done separately for urban and rural districts to better reflect the current urbanization of the YF risk and to account for population density as an important parameter for the whole risk analysis.

#### Qualitative assessment

Following the assignment of district vulnerability based on the above quantitative process, we convened consensus groups to evaluate the results and modify them if necessary, with particular emphasis on identifying additional districts at high risk that were not identified through the quantitative assessment. Consensus group members included staff members of the Ministries of Health, members of nongovernmental organizations with vaccination activities, and international or national YF experts. Consensus groups were instructed to modify the results of the quantitative process to reflect additional local data not included in the quantitative process. These elements included factors such as population migration, main roads linked to districts at risk, resilience of the district, nomadic communities crossing the district, living conditions, and other evidence-based factors that could have an impact on the vulnerability to YF as defined in the vulnerability framework.

## Results

**Figure 5:**
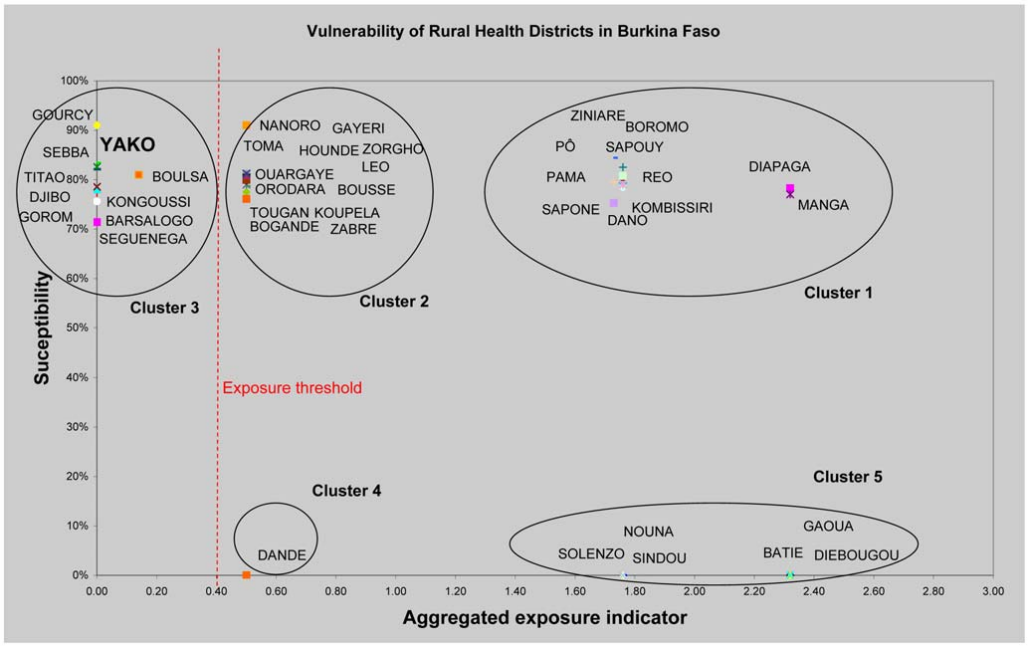
Yellow fever vulnerability of rural districts in Burkina Faso.

The vulnerability graph obtained for rural districts in Burkina Faso illustrates the results of the quantitative analysis (Figure 5).

The rural districts were clustered into five distinct groups. The two clusters of districts on the right of the graph were judged to be very vulnerable (Profile 1, see Methods for definition). Therefore, the threshold for exposure was defined by consensus to be placed at point 0.4 of the exposure axis.

The qualitative process identified additional clusters for inclusion in Profile 1. The district of Yako, although in quadrant III (Profile 3 in Figure 4), was considered to be very vulnerable because of its market-gardening industry and the significant cross-border migration occasioned by this commercial activity. Both characteristics, which were not considered in the exposure variable, were included in the MCA model.

**Figure 6:**
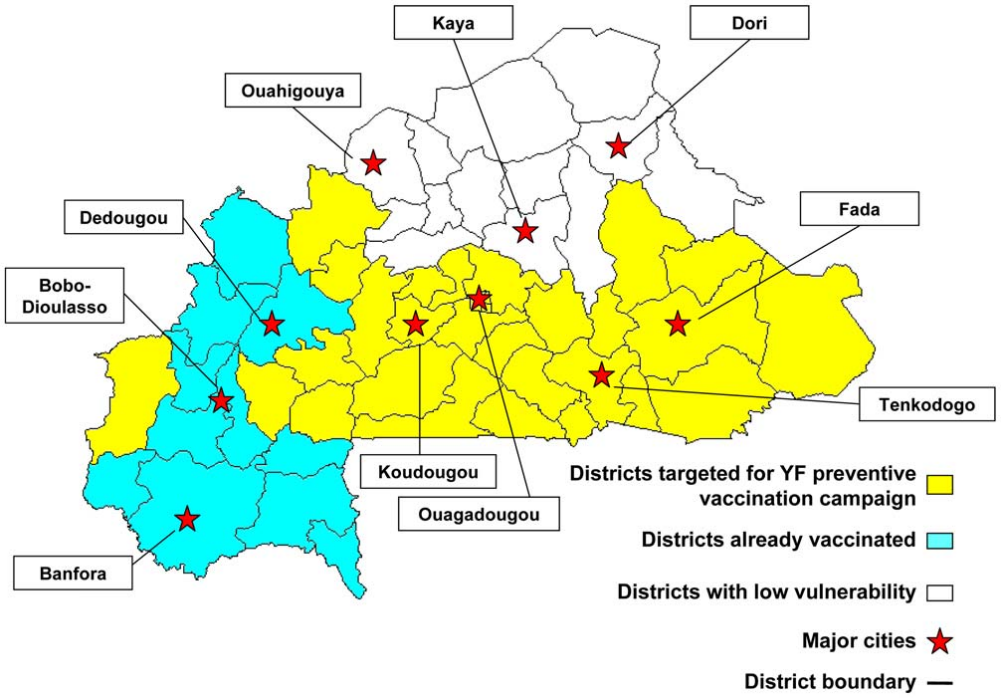
Vulnerability map of all districts in Burkina Faso.

The same analysis was performed separately for urban districts. A map of the vulnerability of rural and urban districts in Burkina Faso is presented (Figure 6). The capital city of Ouagadougou as well as Tenkodogo, Koudougou, and Fada were classified as Profile 1 and will be prioritized for vaccination in the next preventive campaign, whereas the cities of Bobodioulasso, Dedougou, and Banfora were in Profile 2. They have already been vaccinated and they do not need to be revaccinated in the coming years unless the migration rate is known to be high enough to renew the population in a few years time. The other cities, Kaya, Ouhigouya, and Dori, were not priorities for preventive campaigns in the coming years.

On the basis of this analysis a vaccine prioritization schedule was developed including 32 districts out of 55 (see Figure 6) representing 7.8 million people at highest priority for an immediate preventive campaign.

## Discussion

In this article we present the process, which included MCA and consensus group modification, of defining priority communities that would benefit from a YF preventive immunization campaign.

This process starts from a small set of quantitative elements that identify most districts at risk but also engages local decision makers to complement and interpret the results. The MCA allowed the representation of a complex multidimensional situation into a two-dimensional graph that visualizes the communities at highest risk in a concise and reader-friendly way. MCA provides a standardized way to reduce complexity to support an informed decision-making process and allocate effectively the limited resources that are available for this preventive intervention.

The risk assessment is based on the combination of the standardized analysis of defined factors through MCA and information gathered during the consensus meeting that draws on a range of data sources such as epidemic investigation reports, routine surveillance data, or interviews with key personnel in the risk management system. Countries are involved at different steps in the process. The data matrix used for the modeling was verified by the Ministry of Health of Burkina Faso. During the consensus meeting local stakeholders provided information on important risk factors that cannot be handled by the model, for example migratory flows, nomadic populations, and resilience in a particular region. The joint interpretation of the results including the modelers and local stakeholders also provided a better assimilation of the results by the nationals. The full endorsement by the country of the result of the modeling is critical, as the final result of the RA will be translated into the practical implementation of vaccination campaigns, which requires national funding in addition to international financial support. This approach for risk assessment not only supports evidence-based decision making but also empowers decision makers in countries receiving international support for YF control. A criterion for validity of such a process could be the catalytic authenticity which is the extent to which action is stimulated and facilitated by the risk assessment process [59].

One limitation of the described process is that factor analysis such as MCA requires a good dataset with no missing data. The nature of the dataset influences the selection of variables, as mentioned before, and requires control of the quality of the data input into the matrix. The limited amount of information over a long period of time for some indicators was a constraint for integrating more variables into the model. Moreover, the lack of data for some variables, initially considered important during the expert panel meeting, did not allow their final integration into the model. However, the addition of the qualitative consensus review allowed us to overcome this limitation in an efficient manner. The exposure analysis we obtained is similar to the results from other risk mapping studies [14]. The result of the pilot study in Burkina Faso showed that the risk assessment reflects what was instinctively assumed by local health experts. This evidence-based confirmation of subjective knowledge was an important step in the buy-in of political decision makers and the planning of the vaccination campaigns. To date, the risk assessment approach has been used in another seven countries, and results have confirmed the pragmatic approach of this decision-making tool.

A second limitation of the risk assessment tool is the relative (not absolute) characteristics of the aggregated exposure indicator. This limitation means that this value must be compared and interpreted in the frame of one given country and cannot be compared with values calculated from other analyses. If the aggregated exposure indicator equals 1.2 for a district in Burkina Faso, it does not mean that a Togolese district with the same value has the same exposure. This is not a major constraint, as the objective of the YF risk assessment is to rank districts primarily for facilitation of national-level decision-making processes.

The main advantage of multiple correspondence analysis is that the model analyses data without altering the parameters beforehand through scoring procedures and weighting systems. Variables are not weighted before being introduced into the model, but MCA itself, through the comparison of the data of each district, defines the reciprocity of variables for each model outcome. Allowing MCA to define which variables best describe the risk situation for each analysis ensures the highest attainable degree of objectivity. Scoring procedures have often been used for risk assessment using the additive function, with or without weights. Unfortunately, the scoring technique (additive or multiplicative) is unable to discriminate various exposure profiles. For instance, for the YF risk assessment, different values for the five exposure indicators could lead to a similar exposure score. Furthermore, additive scoring procedures often imply managing qualitative variables with subjective assumptions.

This experience shows the robustness of MCA when used with a limited number of variables. It also highlights the potential use of such a methodology for supporting an evidence-based public-health decision-making process in countries where surveillance data of good quality are scarce. A similar methodology based on an original, robust, and reproducible technique—able to give a simple representation of a complex reality—could be used for other infectious diseases such as avian influenza when multiple risk factors at the animal–human interface are interconnected.
